# Differential impacts of pesticides on *Euschistus heros* (Hem.: Pentatomidae) and its parasitoid *Telenomus podisi* (Hym.: Platygastridae)

**DOI:** 10.1038/s41598-019-42975-4

**Published:** 2019-04-25

**Authors:** Juliano de Bastos Pazini, Aline Costa Padilha, Deise Cagliari, Flávio Amaral Bueno, Matheus Rakes, Moisés João Zotti, José Francisco da Silva Martins, Anderson Dionei Grützmacher

**Affiliations:** 1 Federal University of Pelotas (UFPel), Faculty of Agronomy “Eliseu Maciel” (FAEM), Department of Plant Protection (DFs). Campus Universitário, Eliseu Maciel Avenue, P.O. Box 354, Postal Code 96010-900 Pelotas, Rio Grande do Sul, RS Brazil; 2 Brazilian Agricultural Research Corporation (Embrapa), Embrapa Temperate Agriculture. BR 392, km 79, Monte Bonito, P.O. Box 403, Postal Code 96010-971 Pelotas, Rio Grande do Sul, RS Brazil

**Keywords:** Plant sciences, Environmental impact, Plant sciences, Environmental impact, Plant sciences

## Abstract

*Euschistus heros* (Fabricius) (Hemiptera: Pentatomidae) primarily attack the pods and seeds of soybean plants, causing severe economic losses in Neotropical Region, and chemical control is essential to avoid these losses. Thus, insecticides more effective against this pest and less toxic to *Telenomus podisi* Ashmead (Hymenoptera: Platygastridae) - the main biological control agent of *E*. *heros* - should be used. In this report, we studied the differential acute impacts of pesticides used in Brazilian soybean against *E*. *heros* and *T*. *podisi* and evaluated their sublethal effects on the parasitoid to identify effective pesticides towards the pest with less harmful effect to the natural enemy. The LC_50_ of the insecticides to *E*. *heros* ranged from 1.20 to 533.74 ng a.i./cm^2^; the order of toxicity was thiamethoxam + lambda-cyhalothrin > acetamiprid + fenpropathrin > zeta-cypermethrin > acephate > imidacloprid. All pesticides were classified as slightly to moderately toxic to *T*. *podisi* based on the risk quotient. The exposure of *T*. *podisi* females to imidacloprid and the insecticide pre-formulated mixtures reduced the emergence of the offspring parasitoids by up to 40% whereas zeta-cypermethrin and the insecticides pre-formulated mixtures reduced offspring survival. The preferred order of choice of insecticides for the management of *E*. *heros* according to agronomic, toxicological, and environmental feasibility was the following: thiamethoxam + lambda-cyhalothrin > zeta-cypermethrin > acetamiprid + fenpropathrin > acephate > imidacloprid. Our study provides important and pioneer information to select insecticides for effective control of *E*. *heros* with lower impacts on *T*. *podisi*.

## Introduction

Soybean (*Glycine max* L.) (Fabaceae: Phaseoleae) is one of the most economically important leguminous crops worldwide. Brazil is the world’s second-largest producer of soybeans with an estimated production of 115 million tons in the harvest season 2017/18, with USD 32.4 billion in soybean exports^1^. However, the potential productivity of soybeans is usually limited by the occurrence of pest insects during the crop season.

Among the several pests infesting soybeans, the stink bugs are of high relevance due to their high population levels and direct feeding on grains and pods, which can transmit diseases, reducing seed quality^2^. The brown stink bug, *Euschistus heros* (Fabricius) (Hemiptera: Pentatomidae), is the most abundant and prevalent stink bug in soybean of Neotropical Region^3^. Insect populations are managed by frequent spraying of insecticides, and in many situations, the chemical control is the only method capable of effectively avoid economic losses^4^.

The management of *E*. *heros* is based on broad-spectrum insecticides, including organophosphates, pyrethroids, and neonicotinoids^5^, without considering the economic threshold and/or using pesticides tank mixtures^3^. As a result, intensive spraying of insecticides causes several problems, including increased residues in food products, intoxication of users, occurrence of resistant insect populations, resurgence and imbalance of beneficial insects that serve as natural enemies^6–9^. Therefore, the sustainability of soybean crops depends on the development of less hazardous pest management strategies, including biological control and the use of selective agrochemicals^10,11^.

The egg parasitoids of the Platygastridae family are considered the main natural enemies of stink bugs pests (Hemiptera: Pentatomidae) in different crops^12–16^. Parasitoids of stink bugs eggs have been used in 0.03 million hectares of soybean crops in South America in augmentative biological control programs^17^. *Telenomus podisi* Ashmead (Hymenoptera: Platygastridae) is the most efficient parasitoid of *E*. *heros* and *Piezodorus guildinii* (Westwood) (Hemiptera: Pentatomidae) eggs^18^, insects which cause the highest economic losses to soybean crops in Brazil^19^. In Brazil, *T*. *podisi* is found from the Midwest^20^ to the extreme South Regions^21^.

Although biological control is essential for Integrated Pest Management (IPM) by maintaining pest populations below economic threshold levels, chemical control in many circumstances is necessary for effective management of stink bugs and other harmful organisms^22^ present simultaneously in soybean crops. Therefore, the choice of chemicals for pest control in IPM programs should not be based only on the agronomic efficiency (e.g. efficiency in pest control) of the products but also on the lowest impact over the pest natural enemies (e.g. selective pesticide)^23^. To date, information on pesticide selectivity has been disregarded when choosing chemicals to pests control in Brazil, because this information is not easily available to farmers^24^, such as on package leaflet or product labels or even on online pesticides database of the Brazilian Ministry of Agriculture and Food Supply^24,25^. This situation has become even more worrying since Brazil is one of the world leaders in agrochemicals use^26^.

Assessment of the acute toxicity of pesticides towards beneficial arthropods traditionally has relied on the determination of an acute median lethal dose or lethal concentration^27^. Previous studies evaluated the differential acute toxicity of pesticides against the target pests and their natural enemies in different crops with the aim of choosing a pesticide with a high degree of lethal toxicity on pests and minimal non-target lethal toxicity^28–30^. In addition to direct pesticide-induced mortality, the sublethal effects must be considered for a complete impact analysis, helping pesticide choice for IPM^31,32^. However, to the best of our knowledge, no studies to date compared the acute toxicity of pesticides on *E*. *heros* and its main biocontrol agent *T*. *podisi* and the sublethal effects on this egg parasitoid. Thus, the aim of this research was to know the differential impacts of pesticides frequently used in soybean crops in Brazil to the brown stink bug *E*. *heros* and its main parasitoid *T*. *podisi*, and determine the sublethal effects on the parasitoid. Once this data were available, we could select those insecticides that were most effective in controlling the pest and with lowest toxicity to the natural enemy.

## Results

The median lethal concentration (LC_50_) values for acephate, imidacloprid, zeta-cypermethrin, acetamiprid + fenpropathrin, and thiamethoxam + lambda-cyhalothrin after exposure in glass vials were significantly different between *E*. *heros* and *T*. *podisi* (Table 1). For *E*. *heros*, the LC_50_ values range from 1.20 to 533.74 ng of a.i. per cm^2^. The order of acute toxicity (from highest to lowest) was thiamethoxam + lambda-cyhalothrin > acetamiprid + fenpropathrin > zeta-cypermethrin > acephate > imidacloprid (LC_50_ values with overlaps in the 95% confidence intervals were classified as having the same level of toxicity) (Table 1).

**Table 1:** Comparative acute toxicity of acephate, imidacloprid, zeta-cypermethrin, acephate + fenpropathrin, and thiamethoxam + lambda-cyhalothrin (LC_50_ in ng of a.i. per cm^2^) to the soybean brown stink bug *Euschistus heros* and the egg parasitoid *Telenomus podisi*.

Insecticide	Insect	*n*	Slope ± SE	CL_50_*	95% CI	χ^2^
Acephate	*E*.*h*.	750	1,86 ± 0,17	381.06	318.59–456.01	14.48
	*T*.*p*.	400	3.03 ± 0.30	57.43	48.63–67.39	3.15
Imidacloprid	*E*.*h*.	650	0.93 ± 0.09	533.74	386.24–672.92	6.77
	*T*.*p*.	400	4.17 ± 0.59	1.85	1.62–2.08	4.22
Zeta-cypermethrin	*E*.*h*.	500	2.14 ± 0.25	86.98	65.61–113.82	4.69
	*T*.*p*.	400	1.72 ± 0.17	20.38	12.74–30.33	12.57
Acetamiprid + fenpropathrin	*E*.*h*.	400	1.40 ± 0.12	35.62	26.28–47.75	3.43
	*T*.*p*.	300	1.55 ± 0.19	5.79	3.01–8.79	3.57
Thiamethoxam + lambda-cyhalothrin	*E*.*h*.	350	0.88 ± 0.12	1.20	0.55–1.76	1.00
	*T*.*p*.	300	1.66 ± 0.22	0.69	0.46–1.39	3.52

*E*.*h*. = *Euschistus heros*; *T*.*p*. = *Telenomus podisi*; *Values whose confidence intervals (95% CI) do not overlap are considered significantly different.

The LC_50_ values for *T*. *podisi* ranged from 0.69 to 57.43 ng of a.i. per cm^2^, and the order of acute toxicity (from highest to lowest) was thiamethoxam + lambda-cyhalothrin > imidacloprid > acetamiprid + fenpropathrin > zeta-cypermethrin > acephate (LC_50_ values with overlaps in the 95% confidence intervals were classified as having the same level of toxicity) (Table 1).

The risk quotient-based classification (RQ) is shown in Table 2. Acephate, imidacloprid, zeta-cypermethrin, acetamiprid + fenpropathrin, and thiamethoxam + lambda-cyhalothrin were classified as slightly to moderately toxic to *T*. *podisi* (50 < RQ ≤ 2500), with values ranging from 79.55 and 1646.67.

**Table 2:** Risk quotient (RQ) of pesticides used in the control of the brown stink bug *Euschistus heros* on the egg parasitoid *Telenomus podisi*.

Insecticide	LC_50_ (mg a.i. L^−1^)	RQ^a^	C^b^
Acephate	2.48	302.42	2
Imidacloprid	0.08	1500.00	2
Zeta-cypermethrin	0.88	79.55	2
Acetamiprid + fenpropathrin	0.25	375.00	2
Thiamethoxam + lambda-cyhalothrin	0.03	1646.67	2

^a^RQ = registered dose [g a.i. ha^−1^]/CL_50_ for *T. podisi* [mg a.i. L^−1^ - Registered dose for the control of *E*. *heros* in soybean (Table 5); ^b^Categories: 1 = harmless (RQ < 50), 2 = slightly to moderately toxic (50 < RQ ≤ 2500), 3 = toxic or dangerous (RQ > 2500).

*T*. *podisi* females exposed to the LC_50_ of acephate, imidacloprid, acetamiprid + fenpropathrin, and thiamethoxam + lambda-cyhalothrin showed significantly decreased in the percentage of parasitized eggs, by up to 13.42% (H = 22.49, df = 5, *P* = 0.0004) (Table 3). Zeta-cypermethrin did not significantly affect egg parasitism compared to the control treatment. However, all pesticides were classified as harmless according to IOBC classes (E < 30%) to egg parasitism by *T*. *podisi* (Table 3). In contrast, the development of the progeny (F_1_) of *T*. *podisi* was significantly affected by the insecticides (H = 17.11, df = 5, *P* = 0.0004) (Table 3). Imidacloprid, zeta-cypermethrin, acetamiprid + fenpropathrin, and thiamethoxam + lambda-cyhalothrin significantly reduced offspring emergence, whereas acephate did not significantly affect emergence compared to the control treatment. Imidacloprid, acetamiprid + fenpropathrin, and thiamethoxam + lambda-cyhalothrin were classified as slightly harmful (class 2) (30% ≤ E ≤ 79%), with a reduction in adult emergence of up to 40%, whereas acephate and zeta-cypermethrin were classified as harmless (class 1) (E < 30%). However, the insecticides did not significantly reduce the percentage of formed males and females compared with the control treatment (H = 31.46, df = 5, *P* = 0.06) (Table 3).

**Table 3:** Rate of parasitism by females of *Telenomus podisi* (F_0_) exposed to the LC_50_ of acephate, imidacloprid, zeta-cypermethrin, acetamiprid + fenpropathrin, and thiamethoxam + lambda-cyhalothrin, emergence rate and offspring sex ratio (F_1_), and respective toxicity classification.

Insecticide	Parasitism (% ± SE)*	E^a^ [C^#^]	Emergence (% ± SE)*	E^b^ [C^#^]	Sex ratio ± SE*
Acephate	89.87 ± 2.50^b^	7.67 [1]	82.16 ± 3.20^ab^	18.38 [1]	0.81 ± 0.03^a^
Imidacloprid	87.73 ± 2.66^b^	9.86 [1]	54.80 ± 9.99^c^	39.56 [2]	0.89 ± 0.11^a^
Zeta-cypermethrin	96.27 ± 1.26^a^	1.10 [1]	72.48 ± 4.96^bc^	18.69 [1]	0.72 ± 0.07^a^
Acetamiprid + fenpropathrin	84.27 ± 3.85^b^	13.42 [1]	65.85 ± 3.89^c^	38.27 [2]	0.83 ± 0.03^a^
Thiamethoxam + lambda-cyhalothrin	84.53 ± 3.53^b^	13.15 [1]	66.46 ± 5.26^c^	31.78 [2]	0.89 ± 0.07^a^
Control	97.33 ± 1.49^a^	—	87.93 ± 3.12^a^	—	0.91 ± 0.01^a^
CV (%)	12.76	—	33.13	—	9.93
H*	22.49	—	17.11	—	31.46
*P*	0.0004	—	0.0004	—	0.06
df	5	—	5	—	5

*Values followed by the same letter in the column do not differ significantly using the Dunn test (*P* < 0.05); ^a^Reduction of parasitism (%); ^b^Reduction of emergence (%); ^#^IOBC classes: 1 = harmless (E < 30%), 2 = slightly harmful (30% ≤ E ≤ 79%), 3 = moderately harmful (80% ≤ E ≤ 99%), 4 = harmful (E > 99%).

**Figure 1:**
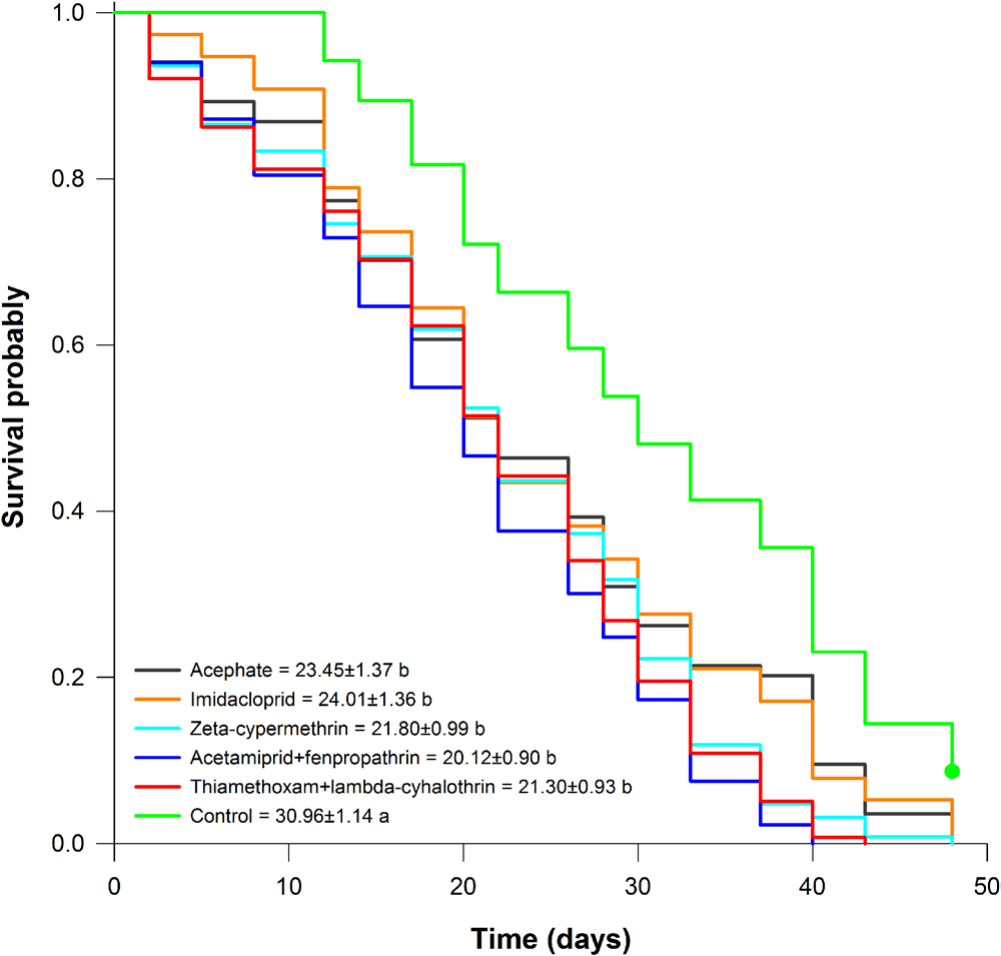
Survival curves for *Telenomus podisi* adults (F_1_) originated from females (F_0_) exposed to LC_50_ of acephate, imidacloprid, zeta-cypermethrin, acetamiprid + fenpropathrin, and thiamethoxam + lambda-cyhalothrin. *The mean survival time (±SE) followed by the same lowercase letter did not differ significantly using the Holm-Sidak test (*P* < 0.05).

The survival of *T*. *podisi* adults originated from females exposed to LC_50_ of acephate, imidacloprid, zeta-cypermethrin, acetamiprid + fenpropathrin, and thiamethoxam + lambda-cyhalothrin was significantly affected (Kaplan-Meier Log-Rank = 68.36, df = 5, *P* =< 0.0001), being observed a decrease of up to 35% compared to the control (Fig. 1). The mean survival of adults (F_1_) was 23.45, 24.01, 21.80, 20.12, and 21.30 days for acephate, imidacloprid, zeta-cypermethrin, acetamiprid + fenpropathrin, and thiamethoxam + lambda-cyhalothrin, respectively. The mean survival in the control group was 30.96 and did not differ significantly from that of acephate and imidacloprid (Fig. 1).

Based on the degree of agronomic, toxicological, and environmental suitability (DA), thiamethoxam + lambda-cyhalothrin was considered the most suitable pesticide to control the brown stink bug (Table 4). The DA order (from highest to lowest) was thiamethoxam + lambda-cyhalothrin > zeta-cypermethrin > acetamiprid + fenpropathrin > acephate > imidacloprid.

**Table 4:** Degree of agronomic, toxicological, and environmental suitability of the insecticides acephate, imidacloprid, zeta-cypermethrin, acephate + fenpropathrin, and thiamethoxam + lambda-cyhalothrin for the control of *Euschistus heros* in soybean.

Insecticide	P^a^	NE^b^		C^c^	EC^d^	SI^e^	DA^f^
		*	**				
	Score^g^						
Acephate	1	5	7	5	3	7	3.7
Imidacloprid	1	5	5	3	5	3	3.0
Zeta-cypermethrin	3	5	7	3	1	7	4.1
Acetamiprid + fenpropathrin	5	5	5	3	1	1	4.0
Thiamethoxam + lambda-cyhalothrin	7	5	5	5	1	1	5.0

^a^Acute toxicity of the insecticide to the pest (P = *E*. *heros*) (Table 1); ^b^Acute toxicity of the insecticide to the natural enemy (NE = *T*. *podisi*) [*classification of the risk quotient (RQ) (Table 2), **IOBC classification of the reduction of parasitism and emergence (Table 3)]; ^b^*RQ < 50 = 7, 50 < QR≤2500 = 5, QR > 2500 = 1; ^b^**E < 30% = 7, 30% ≤ E ≤ 79% = 5, 80% ≤ E ≤ 99% = 3, E > 99% = 1 [score attributed by the highest IOBC toxicity class for parasitism or emergence]; ^c^Toxicological class [package leaflet and label (Table 5)]; ^4^Environmental class [package leaflet and label (Table 5)]; ^c,d^I = 1, II = 3, III = 5, IV = 7; ^e^Safety interval [package leaflet and label (Table 5): ≤ 15 days = 7, 16–20 days = 5, 21–25 days = 3, ≥ 26 days, = 1]; ^f^Degree of adequacy (DA): ≥1 (lower adequacy) to ≤7 (higher adequacy); ^g^Score assigned to original values.

## Discussion

The brown stink bug is one the predominant insect pests in Brazilian soybean crops^6^ and therefore demands high insecticide applications in attempt to regulate population level on the field. These insecticides contain several active ingredients, formulated commercially in isolation or mixtures, presenting different control efficiencies. In this study, *E*. *heros* adults were more susceptible to the residual toxic effects of neonicotinoids and pyrethroids formulated in mixtures, including thiamethoxam + lambda-cyhalothrin and acetamiprid + fenpropathrin, with LC_50_ values of approximately 72, 318, and 445, 2.5, 11, and 15-fold lower than those of zeta-cypermethrin (pyrethroid), acephate (organophosphate), and imidacloprid (neonicotinoid), respectively.

Farmers and phytosanitary managers often prefer to control pest insects in different crops with the same application in tank mixtures^33^ or mixtures of two active ingredients^34^. Spray mixture formulations are registered and currently used to control the brown stink bug in Brazilian soybean crops^25,35^. These pre-mixtures allow a broader spectrum of action, targeting different toxicological sites in the pest. This is due the characteristic of the active ingredients in these mixtures that usually contain a neonicotinoid - systemic in the plant - which acts in the insect mainly by plant tissues/sap ingestion, plus a pyrethroid, which mainly acts by contact. Recent efficiency assays in *E*. *heros* demonstrated a stronger acute effect of commercially formulated neonicotinoids and pyrethroids in mixtures within 14 days after application compared to isolated pesticides such as acephate, zeta-cypermethrin, and imidacloprid^36^.

It is worth highlighting that the concentrations recommended to control *E*. *heros* in the field (ng a.i./cm^2^) are approximately 20, 2, 8, 26, and 409 times higher than the LC_50_ (ng a.i./cm^2^) of acephate, imidacloprid, zeta-cypermethrin, acetamiprid + fenpropathrin, and thiamethoxam + lambda-cyhalothrin, respectively (Supplementary Fig. S1). These results indicate that, in addition to the lower levels necessary to kill 50% of the stink bug population using acetamiprid + fenpropathrin and thiamethoxam + lambda-cyhalothrin, the registered concentrations of these insecticides in the field are much higher than the respective LC_50_ obtained in this study, suggesting the increased safety for the more effective control of *E*. *heros*.

The successful integration of biological and chemical control strategies into an IPM program requires knowledge of the effects of pesticides on beneficial arthropods^23^. For this purpose, several approaches may be used to study the impact of pesticides on natural enemies, including contact exposure or ingestion of toxins using lethal or sublethal doses and field studies to evaluate changes in populations of beneficial insects in response to agrochemical applications^37^.

The results of this study indicated that acephate, imidacloprid, zeta-cypermethrin, acetamiprid + fenpropathrin, and thiamethoxam + lambda-cyhalothrin were toxic to *T*. *podisi*. Organophosphates, neonicotinoids, and pyrethroids impair the synaptic transmission of nerve impulses and axonal neurotransmission by blocking sodium channels, respectively^38^. Neurotoxic insecticides act to a similar extent on different animal phyla, including pest insects and their natural enemies. Therefore, these compounds are more aggressive against egg parasitoids *Telenomus* spp. in different agroecosystems^7,39–41^. Neonicotinoids, pyrethroids and their mixtures presented a lower selectivity to *T*. *podisi*. Similar results were obtained by Turchen *et al*.^41^ and in studies involving the biological control agents *Diadegma* spp. (Hymenoptera: Ichneumonidae) and *Telenomus remus* Nixon (Hymenoptera: Platygastridae)^39,42^.

Adult parasitoids may be directly exposed to insecticide droplets during spraying or indirectly by toxic residues on the plant canopy, water droplets, nectar, or honeydew^43^, and are more sensitive to the effects of pesticides than the immature stages because the embryo is protected by the egg chorion during insect development^44^. Therefore, the RQ was used for the first time to evaluate the ecological risk of insecticide used to control *E*. *heros* over the its natural enemy *T*. *podisi*. The RQ is an important measure of risk to natural enemies under field conditions because it also considers the recommended field rate for target pest control^45^. RQ has been used to evaluate the safety of predators and parasitoids in different agroecosystems^46–49^.

The analysis of the RQ in this study indicated that none of the evaluated pesticides was considered harmless (RQ < 50) to *T*. *podisi*, although the RQ for zeta-cypermethrin approached 50 and was approximately 4, 19, 5, and 20 times lower than that of acephate, imidacloprid, acetamiprid + fenpropathrin, and thiamethoxam + lambda-cyhalothrin, respectively. Acephate and other organophosphates presented a high risk of toxicity to *Trichogramma* spp.^46,48,50,51^. Similarly, neonicotinoids, including imidacloprid and thiamethoxam, were toxic to egg parasitoids^27,46^. Therefore, the use of these insecticides in IPM programs should be carefully evaluated^42,52^.

The exposure of *T*. *podisi* to lethal or sublethal doses of pesticides allows the determination of the chemical and biological compatibility and the effect of insecticides on the natural enemies^31^. Beneficial arthropods surviving insecticide exposure may be mildly or severely affected, manifested in individual biological changes and offspring survival (parasitism rate, adult emergence, longevity/survival, sex ratio), and behavioral characteristics^31,53^.

The exposure to LC_50_ decreased, albeit to a small extent, the percentage of host eggs parasitized by *T*. *podisi*. Previous studies reported significant impairment of parasitism by *Telenomus* spp. and *Trichogramma* spp. exposed to toxic residues at the recommended concentrations of acephate, imidacloprid, zeta-cypermethrin, thiamethoxam + lambda-cyhalothrin, and other organophosphorus pesticides, pyrethroids, and neonicotinoids commercially formulated in isolation or mixtures^7,9,11,22,39,41,45–47,54,55^.

The exposure of *T*. *podisi* females from the maternal generation to insecticides may impair their offspring^53^. In our study, imidacloprid, acetamiprid + fenpropathrin, and thiamethoxam + lambda-cyhalothrin significantly reduced the emergence of offspring of exposed females (Supplementary Fig. S2). In contrast, Bayram *et al*.^40^ evaluated the toxicity of sublethal doses (CL_25_) of the pyrethroids deltamethrin and cyfluthrin to the progeny of females of *Telenomus busseolae* Gahan (Hymenoptera: Platygastridae) and found no detrimental effects on insect emergence. However, studies with parasitoids of the Platygastridae family indicated that insects emergence decreased when immature stages were exposed to pesticides^41,44^. It is of note that our results do not allow determining whether the reduction in insect emergence is due to the direct effects of pesticides or the occurrence of other dysfunctions such as organ malformation^31^.

Sohrabi *et al*.^56^ pointed out that it is vital to consider the fitness of emerging parasitoids. In our study, the survival of the offspring of females exposed to the LC_50_ of zeta-cypermethrin, acetamiprid + fenpropathrin, and thiamethoxam + lambda-cyhalothrin was significantly reduced. The effect of insecticides on the parasitoid longevity will depend on the type of insecticide, parasitoid species, and the mode of insecticide application^40^. For instance, these reductions in longevity are commonly observed in parasitoids emerged from eggs exposed to pesticides while developing inside the host^32,57^. However, Beserra and Parra^53^ found no significant changes in the longevity of F_1_ females of *Trichogramma pretiosum* Riley (Hymenoptera: Trichogrammatidae) developing in eggs of *Anagasta kuehniella* (Zeller) (Lepidoptera: Pyralidae) treated with lambda-cyhalothrin in the larval, pre-pupal, and pupal stages of the parasitoid. Until now, to the best of our knowledge, there are no available studies on changes in the biological characteristics of Platygastridae egg parasitoids, including offspring longevity, as a consequence of the exposure of maternal females to sublethal pesticides concentrations.

Agrochemicals products can also cause changes in the sex ratio of beneficial insects^31^. For instance, the organophosphorus insecticide chlorpyrifos modified the sex ratio of the offspring of several Hymenoptera parasitoid^58,59^, whereas imidacloprid significantly changed the sex ratio of the progeny of *Encarsia inaron* Walker (Hymenoptera: Aphelinidae) by increasing the number of male offspring^56^. However, these authors did not determine the mechanisms underlying the change in the sex ratio of beneficial arthropods caused by insecticides. In the present study, the proportion of F_1_ females remained high in all treatments, and these results are similar to those obtained in a study that determined the rate of parasitism of *E*. *heros* eggs by *T*. *podisi in vitro*^60^.

Bueno *et al*.^23^ recently performed a research review about the challenges, limitations and field recommendations on the selectivity of pesticides to natural enemies, and reported that considering the populations dynamics of insects and other pests and the frequent introduction of new chemical compounds for pest management, farmers need complete information on pesticides that could effectively control target pests with minimal impact on the agroecosystem and related agents to continually adjust their IPM routines. In this research, DA was studied for acephate, imidacloprid, zeta-cypermethrin, acetamiprid + fenpropathrin, and thiamethoxam + lambda-cyhalothrin in order to support the choice of the most appropriate insecticide in an integrated management program for *E*. *heros*. This method was proposed by Martins *et al*.^24^ for pesticides registered in rice, corn, and soybean crops in Southern Brazil. The strong lethal effect of thiamethoxam + lambda-cyhalothrin against *E*. *heros*, with a moderate classification by RQ and sublethal effects on *T*. *podisi*, together with the toxicological class of the commercial product (Table 5), made this insecticide more suitable for pest management.

**Table 5:** Insecticides used in the bioassays of lethal toxicity to the soybean brown bug *Euschistus heros* and lethal and sublethal toxicity to the egg parasitoid *Telenomus podisi*.

Active ingredient (a.i.)	Trade name	Concentration [Formulation]^a^	Registered dose		C^d^	EC^e^	SI^f^	Chemical group
			a.i. ha^−1b^	c.p. ha^−1c^				
Acephate^i^	Orthene 750 BR	750 [SP]	750	1000	III	II	14	Organophosphorus [1B]
Imidacloprid^ii^	Imidacloprid Nortox	480 [SC]	120	250	II	III	21	Neonicotinoid [4 A]
Zeta-cypermethrin^iii^	Mustang 350 EC	350 [EC]	70	200	II	I	15	Pyrethroid [3 A]
Acetamiprid + fenpropathrin^iv^	Bold	75 + 112.5 [EW]	93.75	500	II	I	30	Neonicotinoid [4 A] + Pyrethroid [3 A]
Thiamethoxam + lambda-cyhalothrin^v^	Engeo^TM^ Pleno	141 + 106 [SC]	49.40	200	III	I	30	Neonicotinoid [4 A] + Pyrethroid [3 A]

^i^Arysta Lifescience do Brasil Indústria Química e Agropecuária S/A; ^ii^Nortox S/A; ^iii^FMC Química do Brasil Ltda; ^iv^Iharabras S/A Indústrias Químicas; ^v^Syngenta Proteção de Cultivos Ltda; ^a^Concentration in g a.i./kg or L [EC = emulsifiable concentrate, EW = oil-in-water emulsion, SC = suspension concentrate, SP = water soluble powder]; ^b^Registered dose for the control of *E*. *heros* in soybean crops (Brasil 2018) in g a.i./ha and ^e^g or mL of commercial product (c.p.)/ha; ^d^Toxicological class (package leaflet and label): I = extremely toxic, II = highly toxic, III = moderately toxic, IV = slightly toxic. ^e^Environmental class (package leaflet and label): I = extremely hazardous, II = very hazardous, III = moderately hazardous, IV = slightly hazardous. ^f^Safety interval in days.

It is also worth pointing out that all variables of DA calculation consider the acute toxicity of the pesticides to a pest insect and its natural enemy, because this is essential for practical field applications in order to select the most environment-friendly and less detrimental chemical for pest management. Since the acute toxicity of acephate, imidacloprid, zeta-cypermethrin, acetamiprid + fenpropathrin, and thiamethoxam + lambda-cyhalothrin was high for *T*. *podisi*, these insecticides should be used for the control of *E*. *heros* only in population densities causing economic losses to soybean. Furthermore, future researches to evaluate pesticides sprayed on plant surface and their systemic properties, routes of exposure, metabolism, long-term effects, such as chronic toxicity, bioaccumulation, and bio-magnification can be considered to improve the indication of pesticides for pest management. Even so, in the context of the soybean IPM, the informations obtained in this research are relevant and pioneer in the field of identifying preferred insecticides for the effective control of *E*. *heros* and the preservation of non-target organisms in the soybean agroecosystem.

## Methods

### Insects

The *E*. *heros* colony, originated from adults collected in soybean crop (27°48′1.7352′S, 52°54′3.834″W) in the year 2015, was established by mass rearing in the laboratory [temperature: 25 ± 1 °C; RH: 70 ± 10%; photoperiod: 14:10 (L:D)]^61^. The *T*. *podisi* colony was obtained from “BUG Brasil Agentes Biológicos^©^” and reared in the laboratory [temperature: 25 ± 1 °C; RH: 70 ± 10%; photoperiod: 14:10 (L: D)]^62^.

### Insecticides

Five commercial formulations of insecticides registered for the control of *E*. *heros* in soybean crop^25^ and widely used for managing pentatomids in crop^36^ (Table 5) were used.

### Acute toxicity bioassays

The method using glass vial, initially developed to assess the susceptibility of *Lygus lineolaris* (Palisot de Beauvois) (Hemiptera: Miridae) and *E*. *heros*^63–65^ to contact insecticides, with slight modifications, was used in insecticide toxicity bioassays for *E*. *heros* and *T*. *podisi* in the laboratory [temperature: 25 ± 1 °C; RH: 70 ± 10%; photoperiod: 14:10 (L: D)].

The concentrations of each insecticide used in the assays were based on the level of active ingredient indicated in the package label of the formulations and were prepared in two phases. The first phase consisted of serial dilutions (1:10) of the insecticide stock concentration (1000 ng a.i./cm^2^) to obtain the range of doses causing mortality of 0 to 100%. In the second phase, seven to ten concentrations of each insecticide were prepared by sequential dilution in distilled water to obtain the concentration-response curves and the estimated median lethal concentration (LC_50_). Distilled water was used in the control treatment.

### Toxicity to *E*. *heros*

The surface of each glass vial (2.4 cm in diameter × 8.0 cm in height = 64.84 cm^2^) was impregnated with 600 μL of each insecticide concentration (treatment). The following minimum and maximum concentrations (in ng a.i./cm^2^) were used: acephate (Orthene^®^ SP) 46.27 to 9253.55; imidacloprid (Imidacloprid Nortox^®^ SC) 0.46 to 9253.55; zeta-cypermethrin (Mustang^®^ EC) 0.46 to 9253.55; acetamiprid + fenpropathrin (Bold^®^ EW) 0.09 to 4626.77; and thiamethoxam + lambda-cyhalothrin (Engeo™ Pleno SC) 0.09 to 9253.55. The vials treated were dried on a rotating equipment to ensure the uniformity of mix in the vials. Each treatment included five replicates, each with five pairs (male and female) of stink bugs adults aged ≤72 h.

After 4 h of treatment, the insects were removed from the vials and transferred to small plastic pots (7.0 cm in diameter and 8.5 cm in height) containing beans, soybeans and peanuts as food and water *ad libitum* supplied in 1.5-mL Eppendorf tubes covered with cotton. Insect mortality was evaluated at 24 h and 48 h after contact with the insecticides. The insects that did not move with a stimulus with a fine-tipped brush were considered dead.

### Toxicity to *T*. *podisi*

The application and drying of the insecticides on the surface of the glass vials (1.0 cm in diameter and 8.0 cm in height = 25.91 cm^2^) were performed as described for the *E*. *heros* lethal toxicity bioassay. The following minimum and maximum insecticide concentrations (in ng a.i./cm^2^) were used: acephate (Orthene^®^ SP) 2.32 to 1157.85; imidacloprid (Imidacloprid Nortox^®^ SC) 0.23 to 23.16; zeta-cypermethrin (Mustang^®^ EC) 0.23 to 231.57; acetamiprid + fenpropathrin (Bold^®^ EW) 1.16 to 115.79; and thiamethoxam + lambda-cyhalothrin (Engeo™ Pleno SC) 0.23 to 23.16. Each treatment included five repetitions, each with five pairs (male and female) of parasitoid adults aged ≤48 h.

The parasitoids were removed from the vials after 4 h of treatment and transferred to glass vials (diameter of 2.4 cm and height of 8.0 cm) containing pure honey as food. Mortality was assessed at 24 and 48 h after insecticide exposure. The parasitoids that did not move when stimulated with a fine-tipped brush were considered dead.

### Sublethal effects to *T*. *podisi*

The pairs of *T*. *podisi* were established and maintained for 24–36 h in glass vials (diameter of 2.4 cm and height of 8.0 cm) containing pure honey as food for mating. Subsequently, females (mated, fed, and without foraging experience with the host) were transferred to glass vials (diameter of 1.0 cm, height of 8.0 cm, surface area of 25.91 cm^2^) impregnated with insecticide (LC_50_) or distilled water (control treatment). Five repetitions with 20 females each were used.

After 4 h of treatment with the insecticides (LC_50_), the parasitoids were removed from the glass vials and transferred to another vials (diameter of 2.4 cm and height of 8.0 cm) containing pure honey as food. The mortality ratio was determined 24 and 48 h after contact with the pesticides. Twenty surviving females, randomly selected from each treatment, were transferred to glass vial of the same size containing pure honey as food and an egg mass (aged < 12 h) of *E*. *heros* (cards with approximately 25 eggs) to parasitize for 24 h. The egg cards were removed and individualized to measure the rate of eggs parasitized by the females (generation F_0_) exposed to insecticides (LC_50_), emergence rate, sex ratio and survival of adult parasitoids (F_1_ generation).

### Statistical analysis and toxicity classification

The LC_50_ values, 95% confidence intervals (95% CI), and χ^2^ values were calculated by Probit analysis using the POLO Plus software (Leora Software, Berkeley, CA, USA). The LC_50_ values were compared for each species (*E*. *heros* and *T*. *podisi*) using the LC_50_ confidence intervals, being considered significantly different when these intervals did not overlap.

The risk quotients (RQ) of insecticides were calculated from the values of LC_50_ for *T*. *podisi* and the registered dose for the control of *E*. *heros* in soybean crop (Table 5), according to equation (1)^46^. QR values lower than 50 were considered harmless, values from 50 to 2500 were slightly to moderately toxic and values higher than 2500 were considered toxic or dangerous.

1 $${\rm{RQ}}=(\frac{{\rm{registered}}\,{\rm{dose}}\,[{\rm{g}}\,{\rm{a}}{\rm{.i}}{\rm{.}}\,{{\rm{ha}}}^{-1}]}{{{\rm{LC}}}_{{\rm{50}}}{\rm{for}}\,{\rm{the}}\,{\rm{natural}}\,{\rm{enemy}}\,[\mathrm{mg}\,{\rm{a}}{\rm{.i}}{\rm{.}}\,{L}^{-1}]})$$

The effects of parasitoid exposure (LC_50_) on the rate of parasitized eggs, emergence rate and adult sex ratio (F_1_) were determined by the Kruskal-Wallis test with Dunn post hoc test (*P* < 0.05) using R^®^ software^66^. The Kaplan-Meier estimators (Log-Rank method) were used to evaluate survival (days) of adult parasitoids (F_1_) and the survival curves were compared by the Holm-Sidak test (*P* < 0.05) using the software SigmaPlot version 12.3 (Systat Software, San Jose, CA, USA). In addition, descriptive analysis established by the International Organization for Biological and Integrated Control of Noxious Animals and Plants (IOBC) was conducted using equation (2) to classify the insecticides as follows: class 1: harmless (E < 30%); class 2: slightly harmful (30% ≤ E ≤ 79%); class 3: moderately harmful (80% ≤ E ≤ 99%); class 4: harmful (E > 99%)^67^.

2 $${\rm{E}}=(\frac{{\rm{1}}-{\rm{T}}}{{\rm{C}}})\,\ast \,{\rm{100}}$$

where E is the percentage of reduction in parasitism or emergence, T is the mean rate of parasitism or emergence in the treatment groups, and C is the mean rate of parasitism or emergence in the control groups.

We elaborated an indication of the most adequate insecticides for the control of *E*. *heros*^24^. For this purpose, five variables were used, with different weights [W]: a) toxicity to *E*. *heros* [Wa = 4], based on the differences in LC_50_ (in ng of a.i. per cm^2^) for *E*. *heros*; b) toxicity to *T*. *podisi* based on RQ values^36^ [Wb_1_ = 1.5] and reduction of parasitism and emergence using IOBC criteria^38^ [Wb_2_ = 1.5]; c) toxicological class (package leaflet and insecticide label) [Wc = 1]; d) environmental class (package leaflet and insecticide label) [Wd = 1]; and e) safety interval (package leaflet and insecticide label) [We = 1]. For each item, the scores 1 (lower adequacy), 3, 5, or 7 (higher adequacy) were assigned descriptively. Furthermore, the weighted average indicative of the degree of adequacy (DA) of the commercial insecticide for the control of *E*. *heros* was calculated using equation (3).

3 $${\rm{DA}}=\frac{[(\mathrm{Sa}.\mathrm{Wa})+{(\mathrm{Sb}}_{1}.{{\rm{Wb}}}_{1})+{(\mathrm{Sb}}_{2}.{{\rm{Wb}}}_{2})+(\mathrm{Sc}.\mathrm{Wc})+(\mathrm{Sd}.\mathrm{Wd})+(\mathrm{Se}.\mathrm{We})]}{(\mathrm{Wa}+{\rm{Wb}}+{\rm{Wc}}\,{\rm{Wd}}+\mathrm{We})}$$

where S is the score attributed to the toxicity of the pesticide to the pest (*E*. *heros*) multiplied by weight 4; Sb_1_ is the score attributed to the toxicity of the pesticide to the natural enemy (*T*. *podisi*) according to the RQ multiplied by weight 1.5; Sb_2_ is the score attributed to the toxicity of the pesticide to the natural enemy (*T*. *podisi*) according to the highest IOBC classification to reduce parasitism or emergence multiplied by weight 1.5; Sc is the score assigned to the toxicological class of the pesticide multiplied by weight 1; Sd is the score attributed to the environmental toxicological class of the pesticide multiplied by weight 1; Se is the score assigned to the safety interval of the pesticide multiplied by weight 1.

### Ethical approval

This article does not contain any studies with human participants or vertebrate performed by any of the authors.

## Supplementary information

Supplementary Information

## Data Availability

The authors declare no restrictions on the availability of materials or information.
