# Supplementary material for: Differential impacts of pesticides on Euschistus heros (Hem.: Pentatomidae) and its parasitoid Telenomus podisi (Hym.: Platygastridae)
Source: Sci Rep. 2019 Apr 25;9:6544. doi: 10.1038/s41598-019-42975-4 (PMC6483992; doi:10.1038/s41598-019-42975-4)
Supplement: Supplementary file 1 — Supplementary Information [file 41598_2019_42975_MOESM1_ESM.pdf]

Differential impacts of pesticides on *Euschistus heros* (Hem.: Pentatomidae) and its parasitoid *Telenomus podisi* (Hym.: Platygastridae)

Juliano de Bastos Pazini[1*], Aline Costa Padilha[1], Deise Cagliari[1], Flávio Amaral Bueno[1], Matheus Rakes[1], Moisés João Zotti[1], José Francisco da Silva Martins[2], Anderson Dionei Grützmacher[1]

[1]Federal University of Pelotas (UFPel), Faculty of Agronomy "Eliseu Maciel" (FAEM), Department of Plant Protection (DFs). Campus Universitário, Eliseu Maciel Avenue, P.O. Box 354, Postal Code 96010-900, Pelotas, Rio Grande do Sul, RS, Brazil.

[2]Brazilian Agricultural Research Corporation (Embrapa), Embrapa Temperate Agriculture. BR 392, km 79, Monte Bonito, P.O. Box 403, Postal Code 96010-971, Pelotas, Rio Grande do Sul, RS, Brazil.

[*]Corresponding author:

Juliano de Bastos Pazini

julianopazzini@hotmail.com

+55 53 3275-7376 and +55 55 99934-6181

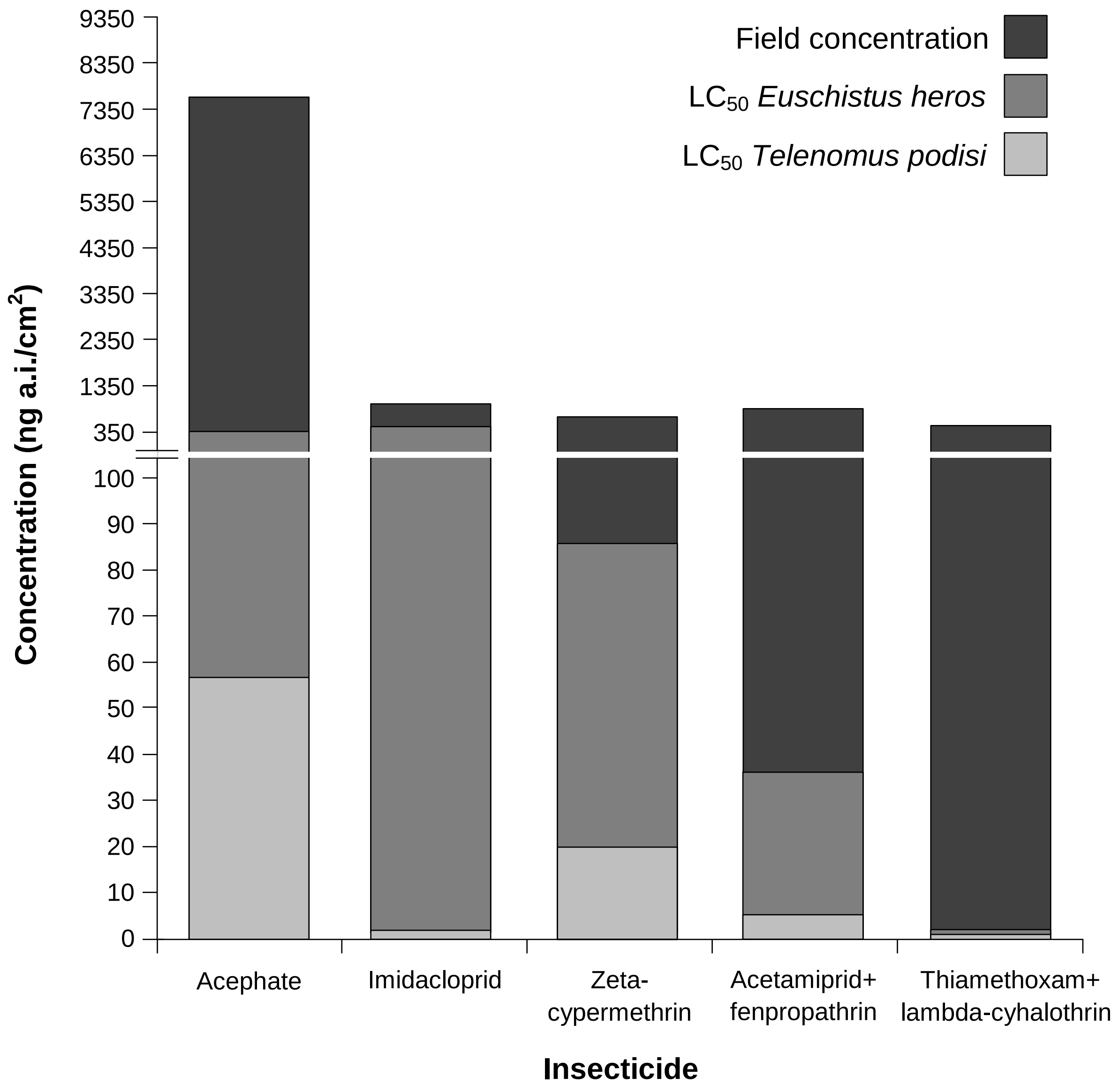

Fig. S1 llustration by column chart highlighting the concentrations recommended for controlling *Euschistus heros* in Brazilian soybean crops (ng a.i./cm^2) and mean lethal concentrations [LC_{50} (ng a.i./cm^2)] for *E. heros* and *Telenomus podisi* to acephate, imidacloprid, zeta-cypermethrin, acetamiprid+fenpropathrin, and thiamethoxam+lambda-cyhalothrin

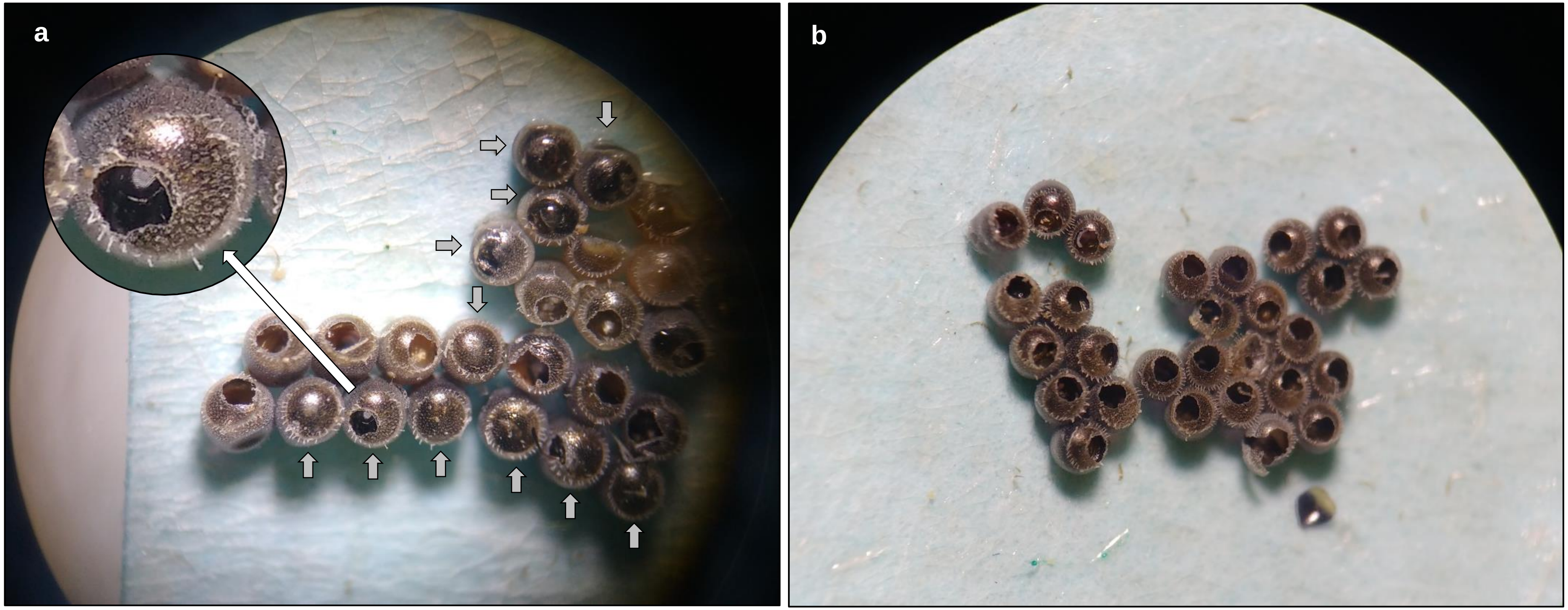

Fig. S2 The exposure of *Telenomus podisi* females from the maternal generation to insecticides impaired their offspring. In our sample, imidacloprid (a) significantly reduced the emergence of offspring of up to 40% compared to the control (b). The gray arrows indicate the adult parasitoids that could not emerge from the eggs. Detail of the parasitoid inside the egg (white arrow)
